# Upcycling agroindustrial waste into graphene oxide supports for gold nanoparticles: toward sustainable nanomaterials

**DOI:** 10.3762/bjnano.17.32

**Published:** 2026-04-01

**Authors:** Juan Marcos Castro-Tapia, Selene Acosta, Hiram Joazet Ojeda-Galván, Elsie Evelyn Araujo-Palomo, Edgar Giovanni Villabona-Leal, Mildred Quintana

**Affiliations:** 1 Centro de Investigación en Ciencias de la Salud y Biomedicina (CICSaB), Av. Sierra Leona 550, Lomas de San Luis, 78210, S.L.P., S.L.P., México; 2 Facultad de Ciencias Químicas, Universidad Autónoma de San Luis Potosí, Av. Dr. Manuel Nava 6, 78210, San Luis Potosí, S.L.P., México; 3 División de Materiales Avanzados, Instituto Potosino de Investigación Científica y Tecnológica A. C., Camino a la Presa de San José 2055, 78216, San Luis Potosí, S.L.P., México; 4 Coordinación para La Innovación y Aplicación de La Ciencia y La Tecnología (CIACYT), Universidad Autónoma de San Luis Potosí, Av. Sierra Leona 550, 78210, San Luis Potosí, S.L.P., México; 5 Departamento de Matemáticas y Física, Instituto Tecnológico y de Estudios Superiores de Occidente, 45601, Anillo Perif. Sur Manuel Gómez Morín 8585, Santa María Tequepexpan, San Pedro Tlaquepaque, Jalisco, México; 6 Facultad de Ciencias, Universidad Autónoma de San Luis Potosí, Av. Parque Chapultepec 1570, Privadas del Pedregal, 78295 San Luis Potosí, S.L.P., México

**Keywords:** agroindustrial waste, gold nanoparticles, graphene oxide, green synthesis, hybrid nanocomposites, plasmonic nanomaterials, surface functionalization

## Abstract

The aim of this work is to develop a sustainable route for producing graphene oxide-based materials from agroindustrial waste and to evaluate their structural and chemical properties in comparison with conventional graphene oxide (GO) and reduced graphene oxide (rGO). Agroindustrial waste-derived graphene oxide (Agro-GO) was synthesized from peanut shells and spent coffee grounds via a ferrocene-assisted green pyrolysis–oxidation approach. The resulting materials were compared with GO prepared via the Marcano-modified Hummers' method and its hydrazine-reduced counterpart (rGO). Comprehensive characterization was performed using UV–vis, ATR-FTIR, XPS, XRD, and Raman measurements, complemented by TGA and TEM to assess optical properties, surface chemistry, and structural and morphological features. Additionally, gold nanoparticles (AuNPs) were photochemically deposited onto Agro-GO to evaluate its potential for nanotechnology-related applications. The results reveal that Agro-GO exhibits tunable oxidation levels, diverse surface functionalities, and morphologies comparable to those of conventional GO; these properties strongly depend on the type of agroindustrial waste precursor used. These findings demonstrate the feasibility of transforming agroindustrial waste into high-value graphene-based nanomaterials and highlight the potential of sustainable synthesis routes for advancing environmentally responsible carbon-based technologies.

## Introduction

Graphene oxide (GO), due to its unique physicochemical properties, is widely explored regarding a range of applications, including sensors, water purification, and energy storage and conversion [1–5]. GO is conventionally synthesized through oxidation of graphite by chemical methods such as the Hummers' method or its modifications, which involve strong acids (e.g., H_2_SO_4_ or H_3_PO_4_), oxidants (e.g., KMnO_4_), and occasionally NaNO_3_ to facilitate exfoliation and oxidation of the graphite layers [6–10]. While effective, these methods have significant drawbacks; they are time-consuming, require substantial energy input, and involve the use of highly reactive and toxic chemical reagents. Additionally, they generate large volumes of hazardous liquid waste, raising environmental and safety risks, especially when scaled up for industrial applications. In this context, there is an urgent need to develop sustainable methods for producing GO-based materials. Alternative approaches such as electrochemical exfoliation, green oxidation processes, hydrothermal treatment, and pyrolysis have emerged as more eco-friendly routes compared to conventional harsh chemical methods [11–14]. In parallel, the increasing demand for eco-friendly, low-cost substitutes has driven the use of biomass-derived carbon precursors, particularly agricultural and food waste, as replacements for graphite [15–17]. These types of waste are abundant, renewable, and often underutilized. Biomass has a high carbon content and aromatic structures, making it suitable for carbonization. This approach not only contributes to waste minimization but also promotes environmental sustainability by converting abundant biowaste into valuable functional materials.

Regarding the pyrolysis of biowaste to obtain GO, most reported approaches rely on high-temperature carbonization, typically at 600–1000 °C for several hours, followed by chemical oxidation [18–20]. For example, Silviana et al. converted coco peat into GO through a two-stage pyrolysis process (350 °C for 1 h and 900 °C for 3 h), producing amorphous carbon that required further oxidation; this route is energy-intensive and generates impurities, making a modified Tour treatment necessary, with the drawback of strong oxidants and substantial chemical waste [18]. Similarly, Sujiono et al. obtained GO from coconut-shell waste by carbonizing it at 600 °C for 3 h and subsequently applying a modified Hummers' method, which still depends on high temperatures and harsh acids such as HF and concentrated H_2_SO_4_ [19]. Mensah et al. produced few-layer GO from birch wood by impregnating the biomass with Mn(NO_3_)_2_ and pyrolyzing it at 900–950 °C to achieve catalytic graphitization. Although this route avoids a separate chemical oxidation step, it still demands prolonged high-temperature treatment [16]. To overcome the limitations of these high-temperature routes, more energy-efficient alternatives based on low-temperature and rapid pyrolysis of biowaste have been proposed [20]. Previous studies have shown that organometallic catalysts can lower the energetic barrier for carbon domain formation, enabling carbonization at temperatures significantly below those required for conventional biomass pyrolysis [21–22]. For instance, Tohamy et al. and Hashmi et al. successfully obtained GO from agroindustrial waste using ferrocene as a catalyst [23–24].

Here, peanut shells and spent coffee grounds were used to produce GO from agroindustrial waste (Agro-GO) via low-temperature, rapid pyrolysis. Peanut shells (*Arachis hypogaea*) are among the main agroindustrial wastes worldwide, with global peanut production reaching 46 million tons in 2019 [25]. These shells are rich in bioactive compounds such as phenolics and flavonoids, as well as cellulose, hemicellulose, and lignin fibers, which are eco-friendly feedstocks for the synthesis of carbon-based materials. Also, the coffee industry, a major global agro-industry, generates over 23 million tons of waste annually from its production and consumption chains [26]. Coffee by-products include husks, pulp, mucilage, parchment, silverskin, and spent coffee grounds; the latter are rich in carbon-containing biomolecules such as cellulose, hemicellulose, lignin, and phenolic compounds, making them ideal as carbonaceous precursors [27–28].

Given that GO is frequently hybridized with other materials to enhance its performance in several applications, it becomes essential to investigate the functionalization potential of Agro-GO materials as viable alternatives to conventionally produced GO [29]. The integration of gold nanoparticles (AuNPs) onto GO yields hybrid materials with synergistic enhancements, such as improved conductivity, plasmonic activity, and high surface area, which significantly boost performance in catalysis, sensing, and energy-related applications [30–33]. Additionally, the deposition of AuNPs onto GO is a practical approach to its surface reactivity, directly linking chemical functionality to nucleation and growth dynamics of AuNPs [34–35].

This study compares GO produced by the modified Hummers' method and its reduced form (rGO) with Agro-GO derived from fast, low-temperature pyrolysis of agroindustrial residues (peanut shells and spent coffee grounds), examining how the carbon source affects the type and content of functional groups and defect density. Ferrocene was used as a catalytic nucleation agent during pyrolysis as its iron-containing framework facilitates the formation of initial aromatic carbon nuclei and promotes early-stage graphitization at low temperatures [20–21]. All GO samples were also used as substrates for AuNP photodeposition to evaluate how their surface chemistry governs nanoparticle nucleation and dispersion. Agro-GO@AuNPs composites demonstrate a green, circular-economy approach with promising potential for applications in sensing, diagnostics, energy storage, and separation technologies.

## Materials and Methods

### Materials

For the synthesis of GO and rGO, all solvents and chemicals were purchased from Sigma-Aldrich and used without further purification. Graphite was purchased from Bay Carbon, Inc.

### Biomass collection and pre-treatment

Peanut shells were obtained from peanuts collected at the Central de Abastos in San Luis Potosí, México. The shells were collected and refrigerated before further processing. Spent coffee grounds were collected from two distinct sources. The first consisted of post-harvest coffee beans from a local coffee farm in Xilitla, San Luis Potosí; the second source comprised commercial ground coffee used in a drip coffee maker at the laboratory, with the grounds recovered immediately after brewing. Both types of waste were vacuum-dried to ensure consistent moisture removal, then milled and passed through a 300 µm mesh sieve to obtain fine particles with a uniform size distribution before pyrolysis.

### Synthesis of graphene oxide

GO was synthesized using the Hummers' method as modified by Marcano and coworkers [9]. Briefly, 3 g of graphite were added to a flask containing a H_2_SO_4_/H_3_PO_4_ mixture, followed by the gradual addition of 18 g of KMnO_4_. The reaction mixture was magnetically stirred at 50 °C for 12 h under reflux. The oxidation process was stopped by adding 3 mL of 30% H_2_O_2_. The resulting mixture was sequentially washed with deionized water, 30% HCl, 99% ethanol and, finally, again with deionized water until reaching a pH of 5. Eventually, GO was coagulated using ethyl ether and dried under vacuum.

### Synthesis of agricultural-waste graphene oxide

Pyrolysis of peanut shells and spent coffee grounds was carried out following the procedure described in [20]. 1 g of the sieved biomass was mixed with 0.1 g of ferrocene, placed in a covered crucible, and heated in air to 300 °C at a heating rate of 10 °C·min^−1^, where it was held for 10 min. The resulting carbonaceous material was recovered and transferred into a 250 mL round-bottom flask containing 60 mL of 50% (v/v) nitric acid to remove residual ionic species introduced by ferrocene. The suspension was stirred at 200 rpm for 12 h at room temperature. Afterwards, the mixture was centrifuged at 7000 rpm for 10 min to precipitate Agro-GO and separate the supernatant containing dissolved iron ions. The solid was washed with deionized water until the pH reached 5. Finally, purified Agro-GO was vacuum-dried for 24 h and ground in an agate mortar to obtain a fine powder suitable for further characterization. The Agro-GO materials derived from peanut shells, coffee waste from Xilitla, and commercial coffee waste are referred to as Agro-GOP, Agro-GOX, and Agro-GOC, respectively.

### Reduction of graphene oxide

The synthesis of rGO was carried out using the GO obtained from section “Synthesis of graphene oxide”. The reduction process was adapted from the methodology reported by Park et al. [36]: 30 mg of GO were dispersed in 10 mL of deionized water and sonicated for 2 h. Subsequently, 10 µL of hydrazine was added, and the mixture was stirred in an oil bath at 80 °C for 3 h. The resulting black powder was recovered by filtration and dried under vacuum to obtain rGO.

### Preparation of the AuNPs@GO hybrids

AuNPs were deposited onto the different synthesized GO materials (GO, rGO, Agro-GOP, Agro-GOX, and Agro-GOC) using a photoreduction method adapted from Hernández-Sánchez and coworkers [30]. 1 mg of the corresponding GO material was dispersed in 80 mL of a 1 M methanol–water solution by ultrasonication. Once the dispersion was stabilized, 58.4 mg of citric acid were added as both reducing and stabilizing agent, and the suspension was stirred magnetically at 220 rpm. Afterwards, the pH was adjusted to 5 by dropwise addition of 1 M NaOH. Then, 400 μL of an aqueous HAuCl_4_ solution (0.05 mM) was introduced into the system. The reaction mixture was irradiated with UV light (GR.E 500 W lamp, Helios Italquartz, ozone-free emission in the 310–450 nm range, λ_max_ = 360 nm) for 60 min under continuous stirring. A purple dispersion was obtained, confirming the formation of AuNPs.

### Characterization techniques

The optical properties of the samples were analyzed by ultraviolet–visible (UV–vis) spectroscopy with a Cary 60 spectrophotometer using 10 mm path-length quartz cuvettes. Measurements were performed using aqueous dispersions (0.5 mg·mL^−1^) previously sonicated for 30 min to ensure homogeneity. Spectra were recorded in the 195–800 nm range. Raman spectra were acquired with a Horiba XploRA Plus micro-Raman spectrometer coupled to an Olympus BX41 optical microscope. The excitation source was a 532 nm solid-state laser (nominal output 28 mW) operated at 25% power using a 20× objective to minimize possible thermal effects on the samples. Spectra were recorded in the 500–3050 cm^−1^ range using a grating of 1800 mm^−1^, with an acquisition time of 30 s and two accumulations, yielding a spectral resolution of 0.8 cm^−1^ per pixel. Attenuated total reflectance Fourier transform infrared (ATR-FTIR) spectroscopy was performed using a Nicolet Nexus 470 FTIR spectrometer (Nicolet, Madison, WI, USA). Spectra were collected at room temperature in the 4000–540 cm^−1^ range with a spectral resolution of 4 cm^−1^, averaging 80 scans per sample. The surface chemical composition was assessed by X-ray photoelectron spectroscopy (XPS) using a SPECS spectrometer equipped with a PHOIBOS 100 energy analyzer and an Al Kα X-ray source (hν = 1486.6 eV). The pass energy was set to 10 eV for high-resolution scans and to 40 eV for survey spectra. All binding energies were referenced to the C=C peak at 284.4 eV. Transmission electron microscopy (TEM) was carried using a JEOL JEM-2100 microscope operating at an accelerating voltage of 200 kV. TEM samples were prepared by drop-casting stable aqueous dispersions of the materials onto carbon-coated copper grids (200 mesh). Thermogravimetric analysis (TGA) was performed using a TA Instruments Q500 thermogravimetric analyzer over a temperature range of 30–700 °C. The heating rate was set at 10 °C·min^−1^, and measurements were carried out under a nitrogen atmosphere with a flow rate of 50 mL·min^−1^. 3 mg of each sample were used for the analysis. X-ray diffraction (XRD) measurements were carried out using a SmartLab RIGAKU diffractometer operating with a Cu anode X-ray source (Cu Kα radiation, λ = 1.5418 Å). The instrument was equipped with a high-speed silicon strip detector (D/teX Ultra). Diffraction patterns were recorded over a 2θ range of 5–80°.

## Results and Discussion

**Figure 1:**
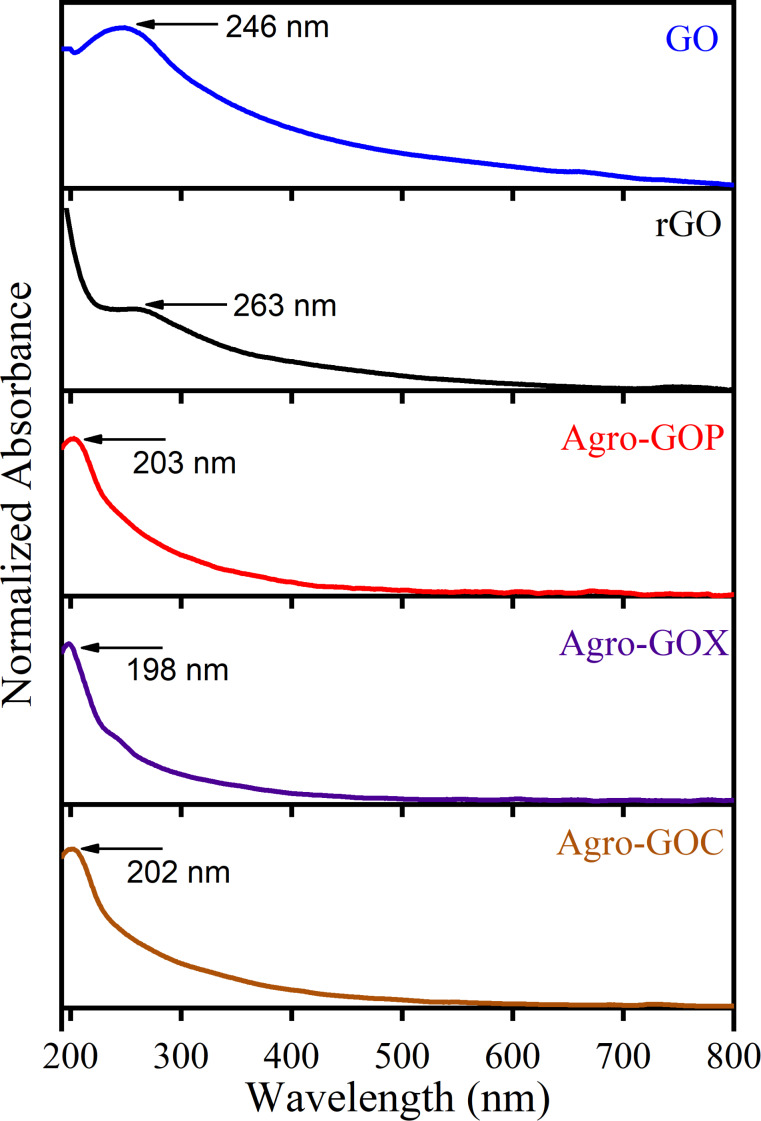
UV–vis absorption spectra of GO samples (GO, rGO, Agro-GOP, Agro-GOX, and Agro-GOC).

Figure 1 shows the UV–vis absorption spectra of the Agro-GOP, Agro-GOX, and Agro-GOC samples compared with the reference materials GO and rGO. This spectroscopy technique provides initial insights into the electronic structure and degree of conjugation of the samples. Typically, the UV–vis absorption spectrum of GO exhibits two characteristic features, namely, a main absorption band in the 230–270 nm region, attributed to the π→π* transition of C=C bonds in sp^2^-hybridized domains, and a shoulder between 300–350 nm corresponding to n→π* transitions of C=O groups in sp^3^-hybridized regions [30]. The position and relative intensity of these features are indicative of the extent of oxidation and disruption of the π-conjugated system within the GO framework. Figure 1 reveals clear distinctions between conventionally synthesized GO, its reduced form rGO, and the Agro-GO materials. GO displays the characteristic π→π* transition near 230 nm and shows the highest absorption in the 300–350 nm region, consistent with the presence of oxygen functionalities responsible for the n→π* transition typically observed in GO. In contrast, rGO presents a redshifted π→π* band at ~270 nm and markedly reduced absorbance in the 300–350 nm region, reflecting partial restoration of conjugated sp^2^ domains and the expected depletion of carbonyl groups upon chemical reduction. Conversely, the Agro-GO samples exhibit a different behavior. All three (Agro-GOP, Agro-GOX, and Agro-GOC) show blueshifted π→π* transitions compared with GO, indicating a significant reduction in the size of the conjugated sp^2^ domains, as smaller and more fragmented sp^2^ clusters require higher excitation energy and, therefore, absorb at shorter wavelengths [37]. Within this series, Agro-GOX stands out by displaying a well-defined absorption band centered around ~244 nm, which lies closer to the range typical of Hummers GO and suggests the presence of somewhat larger or less fragmented sp^2^ domains compared with Agro-GOP and Agro-GOC. Regarding absorption in the 300–350 nm region, Agro-GOC displays comparatively higher absorption than Agro-GOX and Agro-GOP, suggesting a greater abundance of C=O functionalities or more chemically diverse oxygen environments.

**Figure 2:**
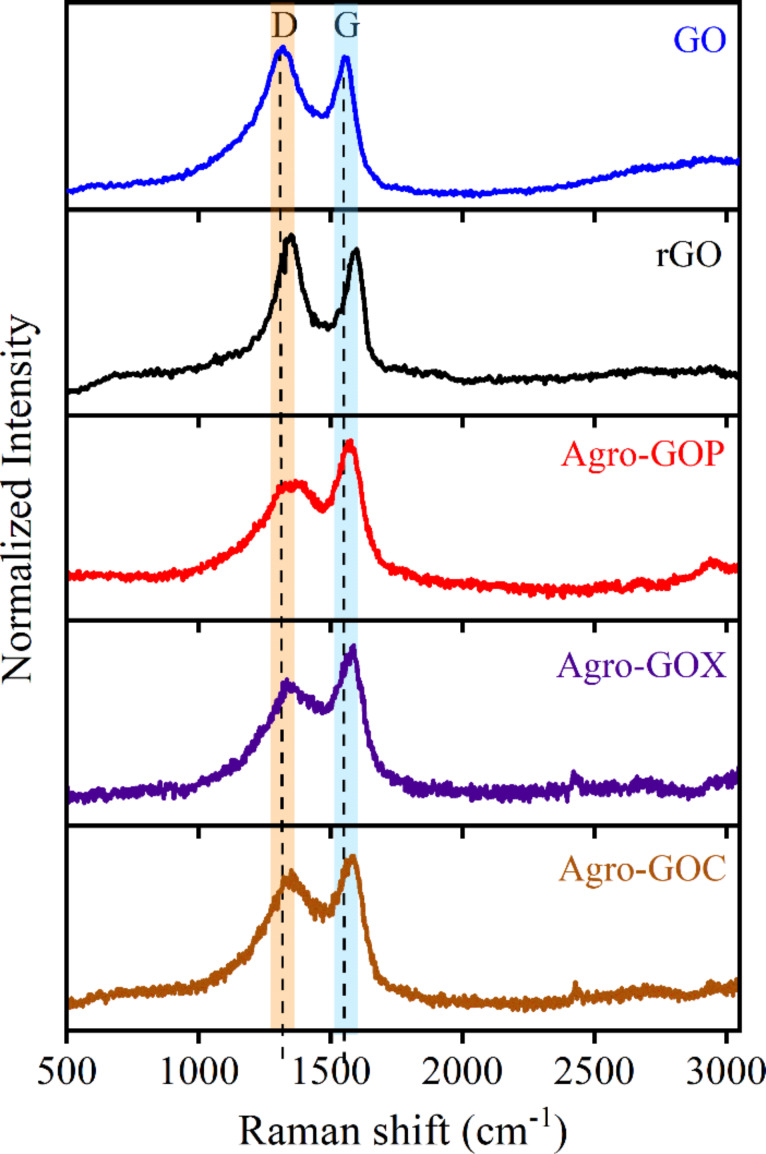
Raman spectra of GO, rGO, Agro-GOP, Agro-GOX, and Agro-GOC samples, highlighting D and G bands.

Raman spectroscopy was employed to further investigate the structural features of the Agro-GO samples (Figure 2). The spectra reveal the two characteristic absorption bands expected for graphene-based materials, that is, the G band located near 1580 cm^−1^, corresponding to the vibrational mode of sp^2^-bonded carbon atoms in aromatic domains and the D band around 1350 cm^−1^, associated with defects in the crystal lattice such as edges, vacancies, and oxygenated functional groups [38]. The intensity ratio between these peaks (*I*_D_/*I*_G_) is commonly used as an indicator of structural disorder or defect density in the material [39]. In the GO spectrum, the D and G bands are observed at 1320.4 and 1555.9 cm^−1^, respectively, with an *I*_D_/*I*_G_ ratio of 1.07. This relatively high ratio indicates a high density of structural defects and a significant degree of oxidation. In contrast, the rGO sample shows a ratio of *I*_D_/*I*_G_ = 1.10; this increased *I*_D_/*I*_G_ ratio compared to GO is commonly reported. Although the reduction process removes oxygen-containing functional groups and partially restores sp^2^-hybridized carbon domains, it also fragments the carbon lattice [40–41]. In contrast, the Agro-GO samples exhibit lower *I*_D_/*I*_G_ ratios of 0.74 (Agro-GOP), 0.78 (Agro-GOX), and 0.91 (Agro-GOC), which at first glance might suggest fewer defects. However, when interpreted alongside the pronounced blueshift of the π→π* transitions in UV–vis spectroscopy, these lower ratios instead indicate that the sp^2^ domains are small and highly fragmented**,** reducing the number of Raman-active defect sites relative to the G-band signal. Such a scenario is typical for highly oxidized, biomass-derived carbons, where the aromatic domains are broken into very small clusters surrounded by abundant oxygen functionalities [42]. Among the Agro-GO materials, Agro-GOC exhibits the highest *I*_D_/*I*_G_ value (0.91), matching its slightly higher absorbance in the 300–350 nm region observed in its UV–vis spectrum. This suggests that Agro-GOC contains a larger fraction of oxygen groups and slightly larger or more defect-rich sp^2^ clusters than Agro-GOP and Agro-GOX. Furthermore, rGO and Agro-GO present a blueshift in the G band. This may be related to phonon stiffening caused by lattice strain and p-type doping; in rGO, this arises from reduction-induced defect restructuring, while, in Agro-GO, it originates from extensive oxidation and the strong confinement of small sp^2^ domains [43–44]. Taken together, the Raman results corroborate the UV–vis spectroscopy analysis by confirming that Agro-GOs possess much smaller and more disrupted sp^2^ domains than GO.

**Figure 3:**
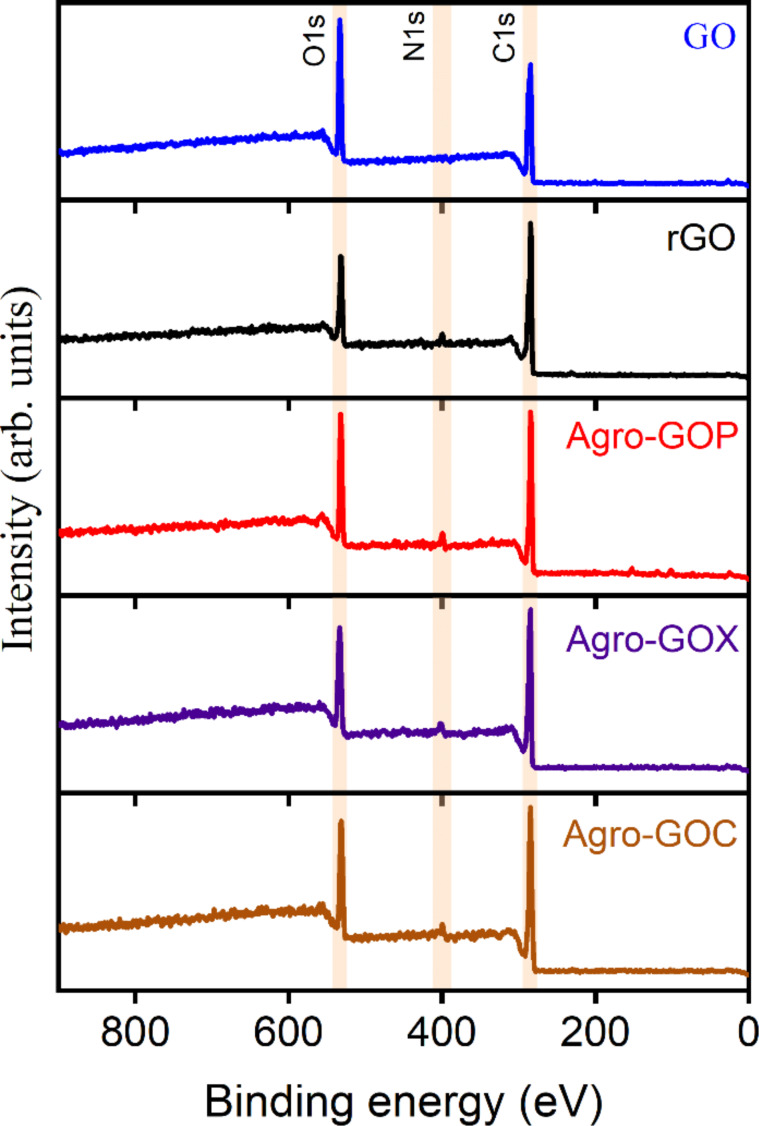
XPS survey spectra of GO, rGO, Agro-GOP, Agro-GOX, and Agro-GOC samples, showing the presence of carbon, oxygen, and nitrogen.

To further elucidate the chemical composition and bonding environments of the samples, XPS was performed. XPS survey spectra were measured to evaluate the surface elemental composition of the synthesized samples (Figure 3). The spectra reveal the presence of carbon and oxygen in all samples, as expected for GO-based materials, along with a minor nitrogen contribution in rGO and the Agro-GO samples. This nitrogen signals in Agro-GOP (4.2%), Agro-GOX (2.7%), and Agro-GOC (2.3%) are attributed to nitrogen-containing compounds, such as amino acids, naturally present in the biomass, which lead to the incorporation of nitrogen functionalities into the Agro-GO samples. In contrast, nitrogen observed in rGO is likely due to the use of hydrazine as a reducing agent, which is likely associated with the formation of –N=C=O or related nitrogen-containing groups [45]. The corresponding relative concentrations (atom %) of carbon, oxygen, and nitrogen in the GO samples are summarized in Table 1. No iron signal was detected, indicating the effective removal of iron residues originated from ferrocene.

**Table 1:** Relative atomic concentrations of the elements detected in the XPS survey spectra for the GO, rGO, Agro-GOP, Agro-GOX, and Agro-GOC samples.

Sample	Carbon [atom %]	Oxygen [atom %]	Nitrogen [atom %]

GO	78.3	22.7	0
rGO	81.6	16.1	2.3
Agro-GOP	75.3	21.5	4.2
Agro-GOX	78.2	19.4	3.4
Agro-GOC	80.1	18.6	2.3

**Figure 4:**
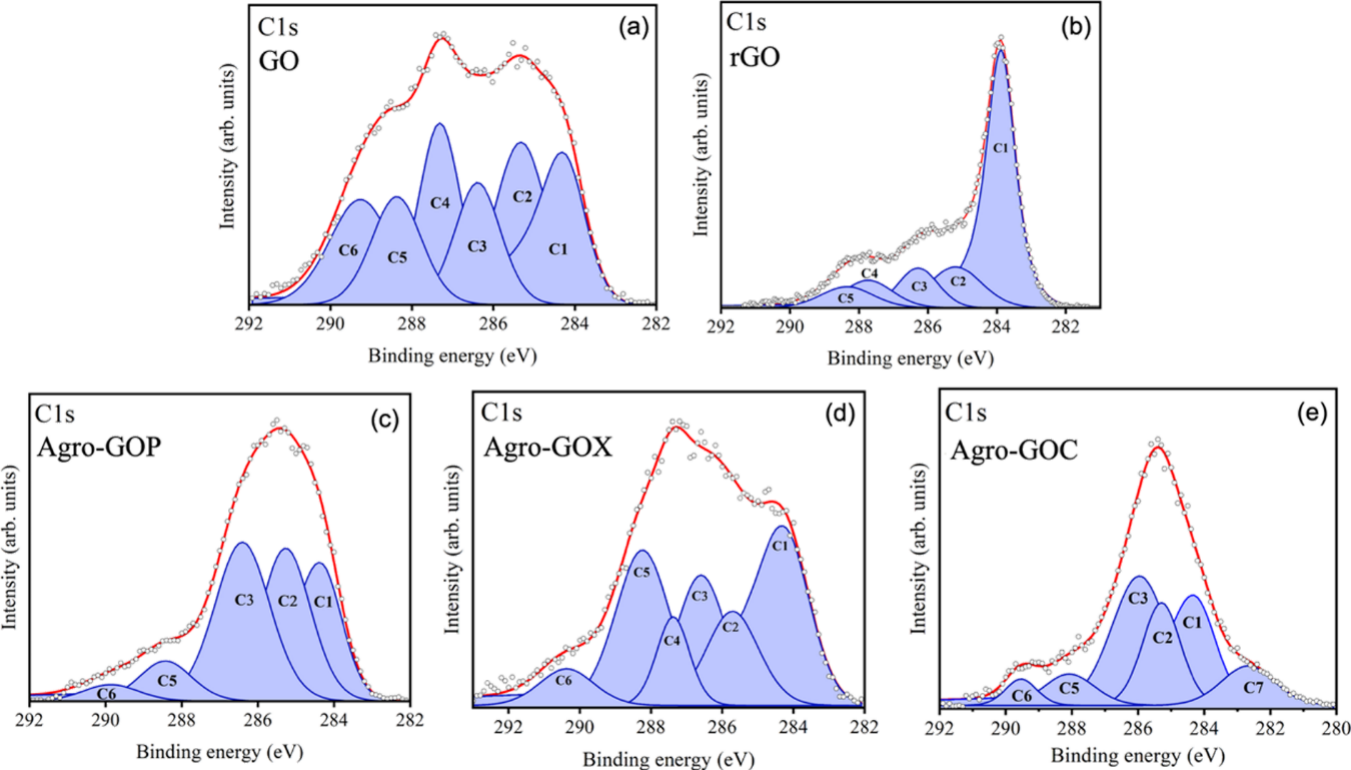
C 1s XPS spectra of (a) GO, (b) rGO, (c) Agro-GOP, (d) Agro-GOX, and (e) Agro-GOC samples. C1 is assigned to sp^2^-C (284.4 eV), C2 to sp^3^-C (285.0 eV), C3 to hydroxy groups C–O (286.4 eV), C4 to epoxy groups C–O–C (287.3 eV), C5 to carbonyl groups C=O (288.4 eV), C6 to carboxyl groups –COOH (289.3 eV), and C7 to vacancy defects (283.0 eV). The components' peaks result from a least-squares fitting procedure.

High-resolution C 1s XPS spectra were deconvoluted to identify the chemical environments of carbon atoms in the GO, rGO, Agro-GOP, Agro-GOX and Agro-GOC samples (Figure 4). The fitted components include C-sp^2^ (sp^2^-hybridized carbon, ~284.4 eV; C1), associated with graphitic domains, C–C (sp^3^-hybridized carbon, ~285.4 eV; C2), attributed to saturated bonds or structural defects, C–O and C–O–C (~286.4–287.3 eV; C3 and C4), corresponding to hydroxy and epoxy groups, respectively, C=O (~288.4 eV; C5), assigned to carbonyl groups, and O–C=O (~289.3 eV; C6), related to carboxylic acid groups [46]. An additional component, C7 (283.0 eV), was required to fit the Agro-GOC spectrum. Such a feature has been reported in graphene-based materials containing significant structural defects, including vacancy-type sites or highly disordered carbon domains [47–48]. The relative concentrations of these components for each sample are summarized in Table 2.

**Table 2:** Relative area (%) of the components used to reproduce the C 1s spectra of the GO, rGO, Agro-GOP, Agro-GOX, and Agro-GOC samples.

Sample	C1 sp^2^-C 284.4 eV	C2 sp^3^-C 285.0 eV	C3 C–O 286.4 eV	C4 C–O–C 287.3 eV	C5 C=O 288.4 eV	C6 COOH 289.3 eV	C7 defects 283.0 eV

GO	32.4	17.0	11.3	15.1	11.0	13.2	0
rGO	63.7	12.6	9.3	6.5	7.9	0	0
Agro-GOP	42.5	22.6	0	26.0	6.2	2.7	0
Agro-GOX	47.8	10.5	13.5	6.2	17.8	4.2	0
Agro-GOC	42.4	14.5	26.5	0	5.8	3.1	7.7

Clear differences in the fraction of sp^2^-hybridized carbon (C1) among the samples are observed. GO contains 32.4% sp^2^-hybridized carbon, reflecting a strongly oxidized GO material in which extended aromatic domains are heavily disrupted, in line with its π→π* band at ~230 nm and relatively high *I*_D_/*I*_G_ ratio (1.07) observed in UV–vis and Raman spectra, respectively (Figure 4a). Chemical reduction markedly increases the sp^2^ fraction in rGO to 63.7% (Figure 4b), confirming partial restoration of graphitic domains; this agrees with the redshifted π→π* transition at ~270 nm and the Raman signature of a more conjugated, yet defect-rich, carbon framework (*I*_D_/*I*_G_ = 1.10). The Agro-GO samples exhibit intermediate C1 values, 42.5% for Agro-GOP (Figure 4c), 47.8% for Agro-GOX (Figure 4d), and 42.4% for Agro-GOC (Figure 4e), indicating that they contain a larger overall fraction of sp^2^-hybridized carbon than GO, but distributed in much smaller and more fragmented domains, as evidenced by the strong blueshift of the π→π* band and the lower *I*_D_/*I*_G_ ratios for Agro-GOP (0.74) and Agro-GOX (0.78). In Agro-GOC, the combination of a moderate sp^2^ fraction (42.4%), the highest *I*_D_/*I*_G_ ratio (0.91), and the presence of the C7 component (7.7%), assigned to highly defective carbon such as vacancy-related sites, supports a picture of more defect-rich sp^2^ clusters, consistent with its enhanced absorbance in the 300–350 nm range. Overall, the XPS-derived sp^2^ fractions corroborate the spectroscopic view that rGO hosts the most extended conjugated network, GO the most oxidized conventional GO, and Agro-GO materials a high but finely partitioned sp^2^ content constrained into small and defective domains.

The relative proportions of oxygen-containing groups obtained from the C 1s deconvolution spectra reveal marked differences among GO, rGO, and Agro-GO samples, indicating that both the synthesis route and the biomass precursor tightly control surface functionalization. In GO, the oxygenated components are balanced, with comparable contributions from C–O (C3, 11.3%), epoxide C–O–C (C4, 15.1%), carbonyl C=O (C5, 11.0%), and carboxyl –COOH (C6, 13.2%) (Figure 4a), which is characteristic of conventional GO produced by strong oxidation of graphite. After chemical reduction, rGO shows a global decrease in oxygenated carbon (C3–C5) and a complete loss of the carboxyl component (C6), consistent with the partial restoration of the graphitic network and removal of the most labile oxygen functionalities (Figure 4b). Conversely, the Agro-GO materials exhibit distinct oxygen-group distributions that depend on the type of biomass used. Agro-GOP is clearly epoxide-rich, with a dominant C4 contribution (26.0%) and no detectable hydroxy C–O (C3 = 0%), suggesting that, under the chosen conditions, epoxide formation is favored, possibly through dehydration of primary C–OH groups initially present in lignocellulosic structures (Figure 4c). Agro-GOX is carbonyl-rich, showing the highest C=O fraction (C5, 17.8%) together with moderate C–O (13.5%) and smaller epoxide and carboxyl contributions. This result points to oxidation pathways that stabilize conjugated carbonyl species, likely influenced by the specific composition of the coffee biomass (e.g., polyphenols, and lipids) (Figure 4d). Finally, Agro-GOC is hydroxy-dominated, with a large C–O contribution (C3, 26.5%) and no epoxide (C4, 0%), indicating a surface enriched in alcohol/ether-type functionalities (Figure 4e). These trends demonstrate that, beyond the overall oxidation level, the nature of the oxygen functionalities can be tuned by selecting different agroindustrial residues as precursors.

Regarding the nitrogen content, although small amounts of nitrogen were detected in some samples, especially the Agro-GO series, nitrogen-containing functional groups could not be clearly resolved in the C 1s spectra due to their binding energies being considerably close to those of oxygenated species. As a result, nitrogen-related contributions were not deconvoluted separately. However, their presence is supported by survey spectra, which indicate that some nitrogen functionalities are indeed incorporated into Agro-GO. The high-resolution O 1s spectra of the GO samples are shown in Figure S1 (Supporting Information File 1), and the relative contributions of the deconvoluted components are summarized in Table S1 (Supporting Information File 1).

**Figure 5:**
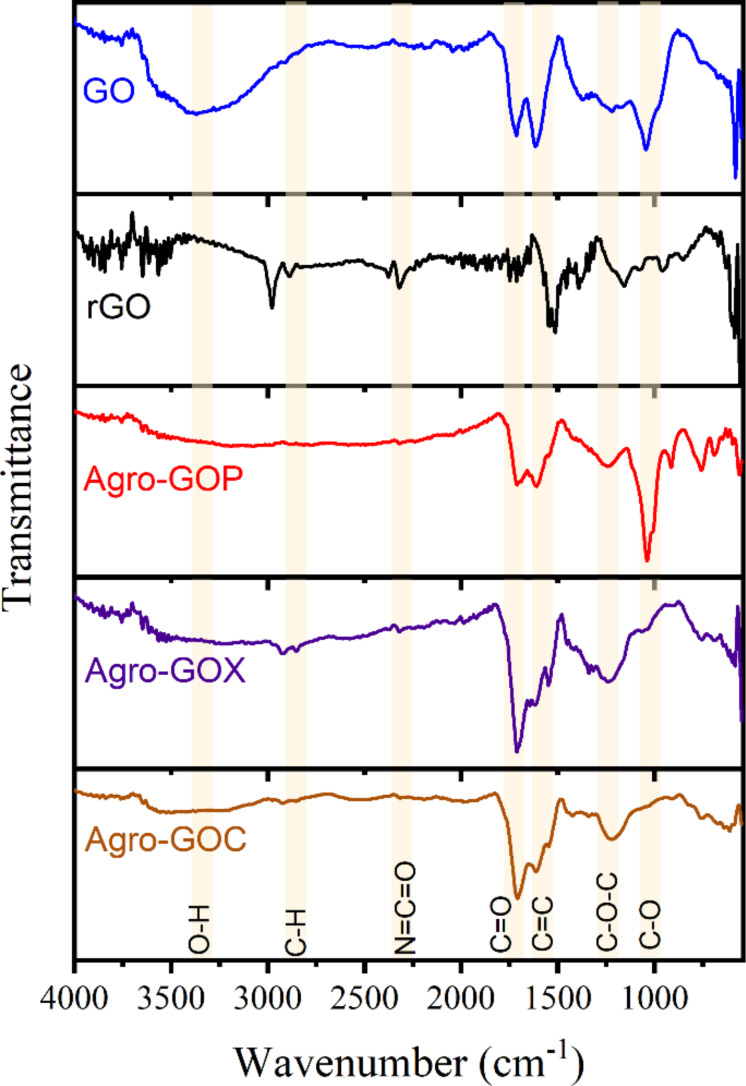
ATR-FTIR spectra of GO samples (GO, rGO, Agro-GOP, Agro-GOX, Agro-GOC), showing characteristic vibrational bands of hydroxy (O–H), aliphatic (C–H), carbonyl (C=O), aromatic (C=C), carboxyl (O=C–O), epoxy (C–O–C) and ether (C–O) groups. The presence of isocyanate (–N=C=O) bands is also noted.

The ATR-FTIR spectra of GO, rGO, Agro-GOP, Agro-GOX, and Agro-GOC samples are shown in Figure 5. The O–H stretching band is observed between 3200 and 3600 cm^−1^, together with weaker aliphatic C–H stretching features near 2850–2950 cm^−1^. The strong band at 1700–1750 cm^−1^, is assigned to carbonyl and carboxyl C=O stretching, and the C=C band near 1580–1620 cm^−1^, reflects sp^2^ aromatic domains [49–50]. The C=C stretching is clearly observed across the spectra, although with varying relative intensities that correlate with oxidation degree and domain fragmentation. GO presents a well-balanced distribution of C–O, epoxy, carbonyl, and carboxyl vibrations, consistent with the oxygen functionalities expected for conventionally synthesized GO, in line with XPS analysis. In contrast, rGO shows attenuated C–O and C=O bands and a relatively more pronounced C=C contribution, reflecting partial deoxygenation and partial restoration of the sp^2^ network. The loss of C–O, C=O, and –COOH species reduces the number and intensity of IR-active vibrational modes, resulting in lower overall absorbance and a lower signal-to-noise ratio. The Agro-GO samples, however, display more heterogeneous and precursor-dependent profiles: Agro-GOP is dominated by epoxy-related bands in the 1200–1300 cm^−1^ region, matching its epoxide-rich composition observed by XPS. Agro-GOX shows the strongest C=O contribution, consistent with its carbonyl-rich XPS signature, and Agro-GOC exhibits intense C–O bands (1000–1100 cm^−1^) and a broad O–H feature, supporting its hydroxy-rich surface chemistry and high defect density. Notably, a band near 2300 cm^−1^ appears in rGO and the Agro-GO samples, which can be attributed to nitrogen-containing groups such as N=C=O or C≡N, in agreement with the nitrogen detected by XPS [51].

**Figure 6:**
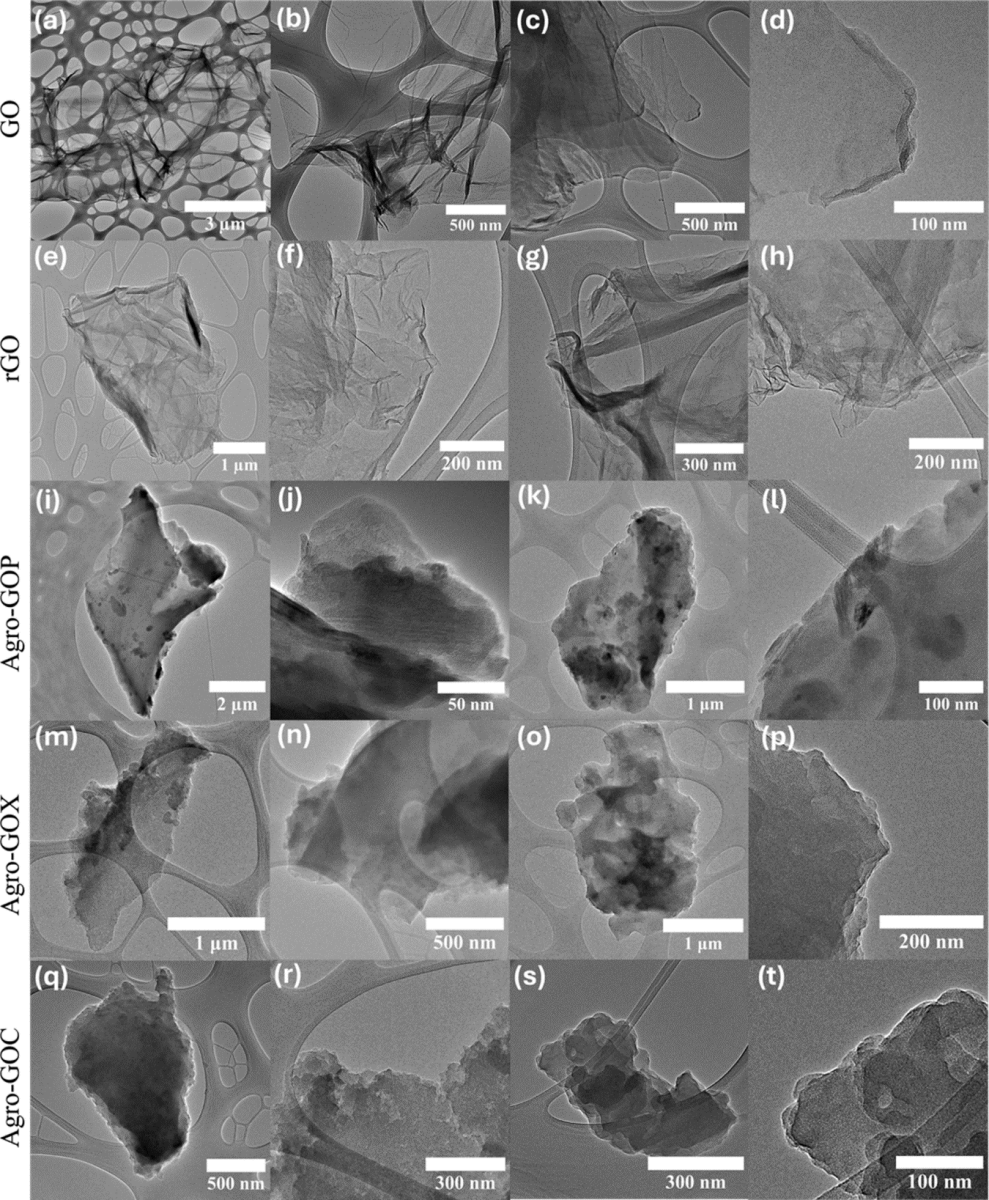
TEM images of GO-based materials: (a–d) GO, (e–h) rGO, (i–l) Agro-GOP, (m–p) Agro-GOX, and (q–t) Agro-GOC.

Figure 6 shows TEM images of GO, rGO, and Agro-GO samples; each row corresponds to a specific sample. The images reveal clear morphological differences among the graphene-based materials depending on their synthesis route. The GO sample (Figure 6a–d) exhibits thin, translucent, and wrinkled sheets with minimal agglomeration, which reflects a high oxidation level and effective exfoliation of the graphite precursor, consistent with typical GO morphology. In contrast, the rGO sample (Figure 6e–h) displays darker, less transparent sheets, with noticeable regions that are flatter and less wrinkled than in GO. These smoother areas corroborate the presence of larger sp^2^ domains resulting from the reduction process. Therefore, there is an increased tendency for sheet restacking due to stronger π–π interactions between the basal planes. Regarding the Agro-GO samples, Agro-GOP (Figure 6i–l) exhibits relatively thick, strongly aggregated sheets with a rough and compact morphology. The lamellae are well defined and contain regions that clearly display stacked sp^2^-hybridized carbon sheets with locally ordered domains that evidence the presence of few-layer graphitic structures. However, regions of amorphous carbon are also observed, consistent with the Raman and XPS results that indicate substantial structural disorder and a significant contribution from oxygenated functional groups. Agro-GOX (Figure 6m–p) displays a more homogeneous dispersion and fewer heavily aggregated regions compared with Agro-GOP. The sheets appear somewhat smoother and more sheet-like, although they still show evidence of partial structural disorder along their edges and within the basal planes. This intermediate morphology matches the spectroscopic observations, which point to a moderate defect density and a balance between disordered and graphitic domains. Agro-GOC (Figure 6q–t) shows the most compact and aggregated morphology, with densely packed, coarse particles and limited evidence of well-separated, few-layer sheets. These observations are consistent with the C 1s XPS analysis, which shows the appearance of the low-binding-energy C7 component, indicating the presence of vacancy-type defects and highly disordered carbon domains.

**Figure 7:**
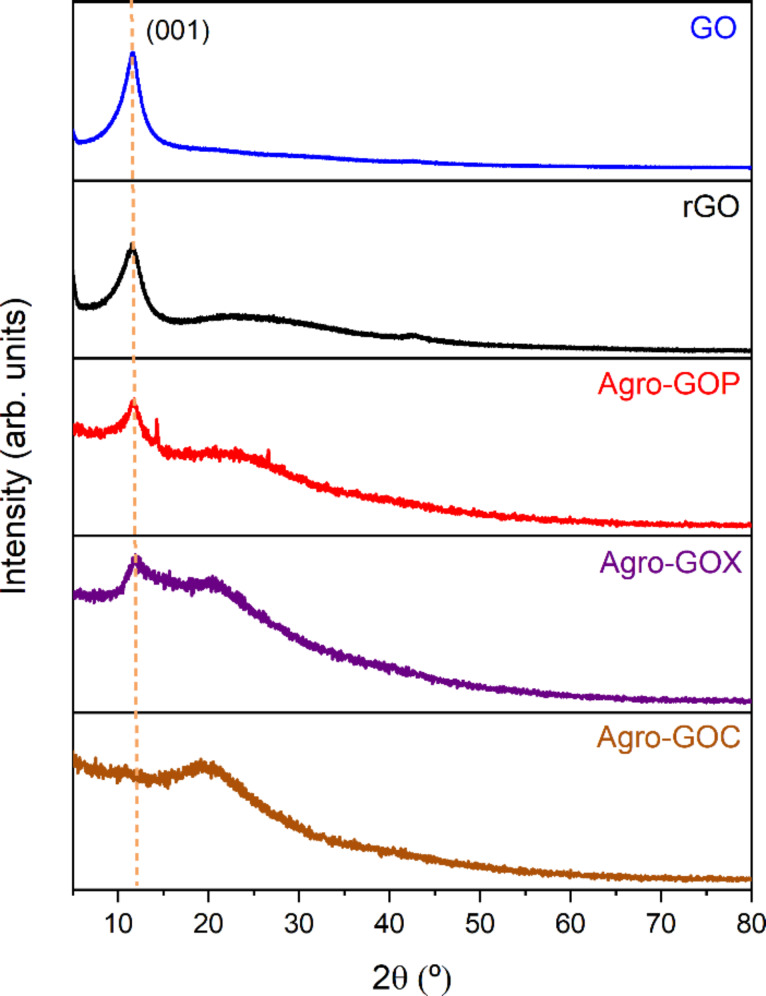
X-ray diffraction (XRD) patterns of GO, rGO, and agroindustrial waste-derived samples (Agro-GOP, Agro-GOX, and Agro-GOC).

X-ray diffraction patterns of the samples are shown in Figure 7. GO exhibits an intense peak at 2θ ≈ 11.7°, assigned to the (001) reflection of GO and associated with an expanded interlayer spacing (*d* ≈ 0.76 nm) due to the presence of oxygen-containing functional groups and intercalated species [9]. In rGO, the (001) reflection decreases in intensity and becomes broader, while a broad contribution centered at ~22.4° appears, suggesting partial reduction and disordered restacking of the sheets. Similarly, the Agro-GOP and Agro-GOX samples retain a maximum near 11–12° (*d* ≈ 0.74–0.76 nm), indicating the presence of GO-like domains. These samples also display a broad diffraction peak at ~19–20°, attributable to poorly ordered carbon. In contrast, Agro-GOC is dominated by a broad diffraction peak at ~19° and lacks a well-defined (001) peak, indicating reduced lamellar periodicity and a predominantly disordered structure, matching the results observed in TEM and XPS.

**Figure 8:**
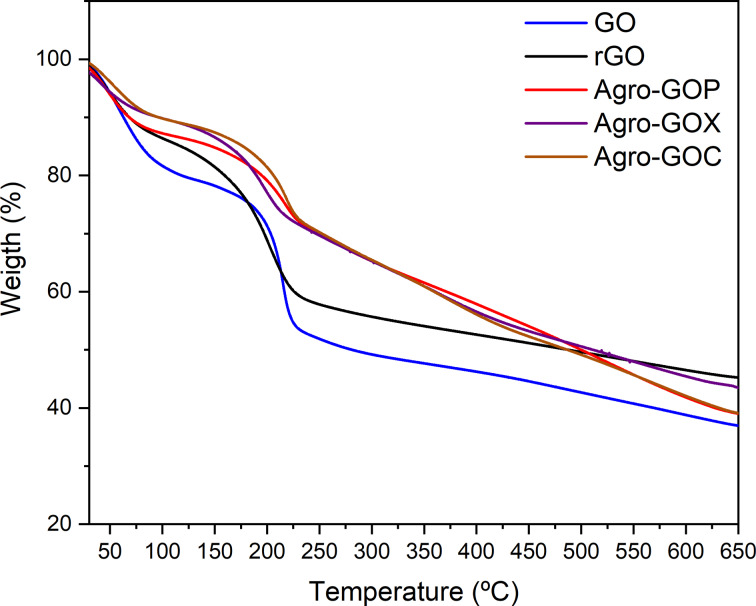
Thermogravimetric analysis (TGA) curves of GO, rGO, and agroindustrial waste-derived samples (Agro-GOP, Agro-GOX, and Agro-GOC).

TGA curves for the samples are shown in Figure 8, exhibiting a characteristic multistep weight-loss behavior typical of oxygenated carbon materials. An initial mass loss below ~100 °C is observed for all samples and is attributed to the removal of physically adsorbed and intercalated water, being more pronounced for GO due to its higher hydrophilicity. GO displays a sharp weight loss in the 150–250 °C range, corresponding to the decomposition of labile oxygen-containing functional groups, whereas rGO shows a less intense and more gradual mass loss, consistent with partial reduction and decreased oxygen content. The Agro-GOP, Agro-GOX, and Agro-GOC samples exhibit intermediate behavior, with moderate weight loss between 150 and 300 °C followed by gradual degradation up to 650 °C, indicating the coexistence of oxidized graphitic domains and amorphous carbon. At higher temperatures (≥600 °C), the agroindustrial waste-derived samples retain a slightly higher residual mass than GO, consistent with the presence of more thermally stable carbon fractions derived from the biomass precursors. Overall, these thermal stability trends are in good agreement with XRD, XPS, Raman, UV–Vis, and TEM characterizations, collectively supporting the coexistence of GO-like domains and amorphous carbon in the agroindustrial waste-derived materials. The corresponding derivative thermogravimetric (DTG) curves are provided in Figure S2 (Supporting Information File 1).

Recent literature demonstrates that biomass-derived GO materials can be obtained via distinct synthetic routes, each yielding different balances among graphitic ordering, defect density, and oxygen functionalization. A common strategy involves high-temperature carbonization (650–950 °C) followed by modified Hummers' oxidation, as reported for tea waste, cashew shell, and coconut shell precursors, in which structural ordering is established during pyrolysis and oxygen functionalities are predominantly introduced during permanganate-based oxidation [19,52–54]. In contrast, the Agro-GO materials presented here are synthesized under substantially milder thermal conditions without a Hummers step. Despite the lower processing temperature, they retain characteristic GO-like features via ferrocene-assisted aromatization, while enabling precursor-dependent tuning of oxygen functionality and sp^2^ domains. Another approach relies on high-temperature catalytic graphitization followed by mild exfoliation, producing few-layer, weakly oxidized GO-like materials; for example, Mn-assisted graphitization at ~950 °C combined with mechanochemical exfoliation yields layer sheets with limited oxidation compared to Hummers-derived GO [16]. Compared to these systems, Agro-GO obtained in this work maintains significant oxygen functionality while preserving fragmented sp^2^ domains, situating it between highly oxidized Hummers-type GO and minimally oxidized graphene-like sheets. Recent ferrocene-assisted low-temperature routes (≈300 °C) further demonstrate the direct conversion of agroindustrial waste into GO-like frameworks without a separate oxidation step [20,23–24]. While these studies establish the feasibility of catalytic low-temperature synthesis across different residues, the present work extends this framework by directly benchmarking Agro-GO against GO and rGO prepared via the Hummers' method and by explicitly correlating oxidation degree and sp^2^-hybridized carbon fraction with biomass precursor. This integrated comparison provides clearer insight into how precursor composition and synthesis pathway govern oxygen functionality, structural disorder, and graphitic domain organization in biomass-derived GO materials.

**Figure 9:**
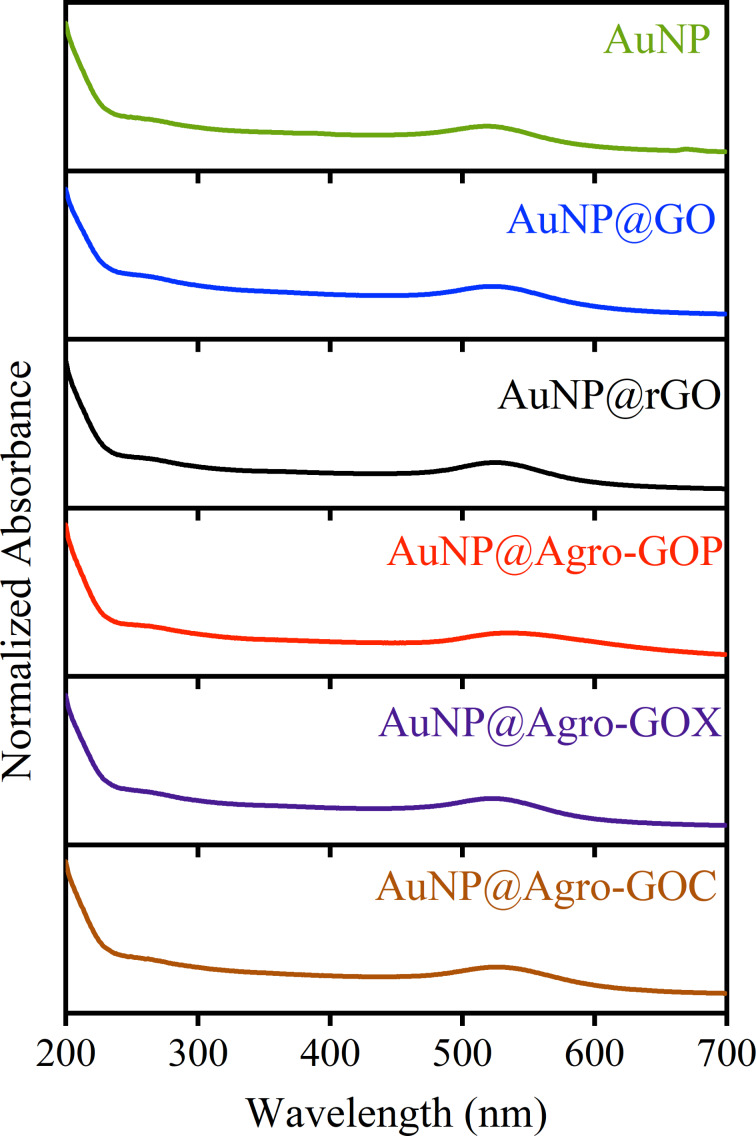
UV–vis spectra of AuNPs and AuNPs supported on GO samples. The plasmon resonance peak appears at 522 nm for AuNPs and is redshifted (526–531 nm) in the hybrids, indicating interaction with the GO surface.

To evaluate the functional performance of the Agro-GO samples, they were employed as platforms for the in situ photochemical growth of AuNPs. This approach takes advantage of the strong affinity between gold precursors and oxygen-containing functional groups on the GO surface, which act as nucleation and anchoring sites for AuNPs, thereby modulating their size, spatial distribution, and interaction with the carbon framework [30,55–56]. For clarity, the resulting hybrids are denoted as AuNP@GO, where the suffix identifies the specific type of GO support. The UV–vis spectra shown in Figure 9 revealed characteristic optical features from both AuNPs and the GO supports. Free AuNPs displayed a localized surface plasmon resonance (LSPR) band centered at 522 nm, indicative of small and well-dispersed AuNPs in colloidal suspension. AuNPs grown on GO substrates showed redshifted LSPR bands in the range of 526–531 nm, accompanied by notable spectral broadening. These features are attributed to interactions between AuNPs and oxygen-containing functional groups on the GO surface, which influence the local dielectric environment and stabilize the AuNPs. The most pronounced redshifts and spectral broadening were observed for the AuNP@Agro-GOX and AuNP@Agro-GOC hybrids, suggesting stronger interfacial interactions and higher surface reactivity.

**Figure 10:**
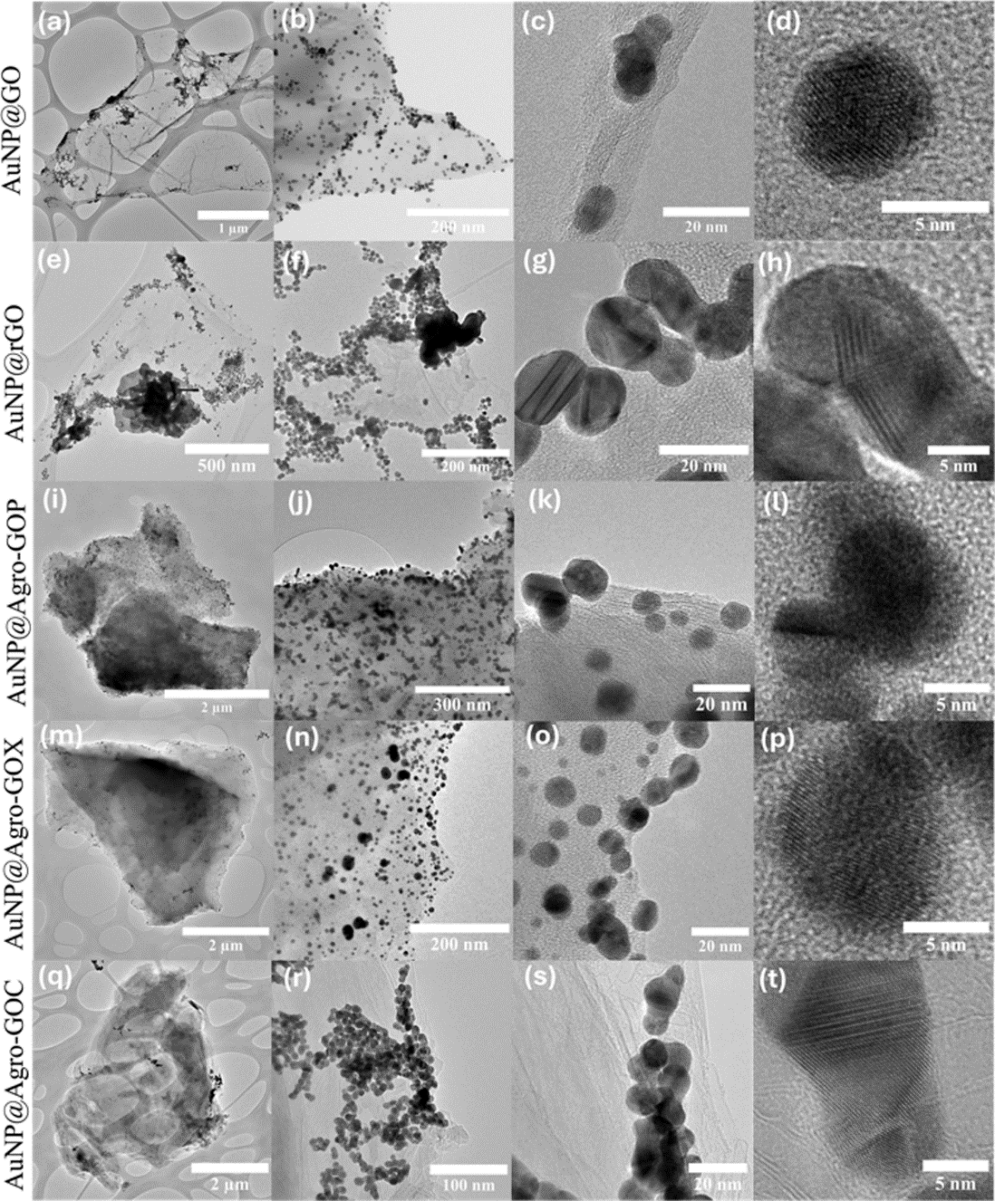
TEM images of AuNP-decorated GO-based materials. Each row represents a hybrid: (a–d) AuNPs@GO, (e–h) AuNPs@rGO, (i–l) AuNPs@Agro-GOP, (m–p) AuNPs@Agro-GOX, and (q–t) AuNPs@Agro-GOC. Columns show increasing magnifications of each sample.

**Figure 11:**
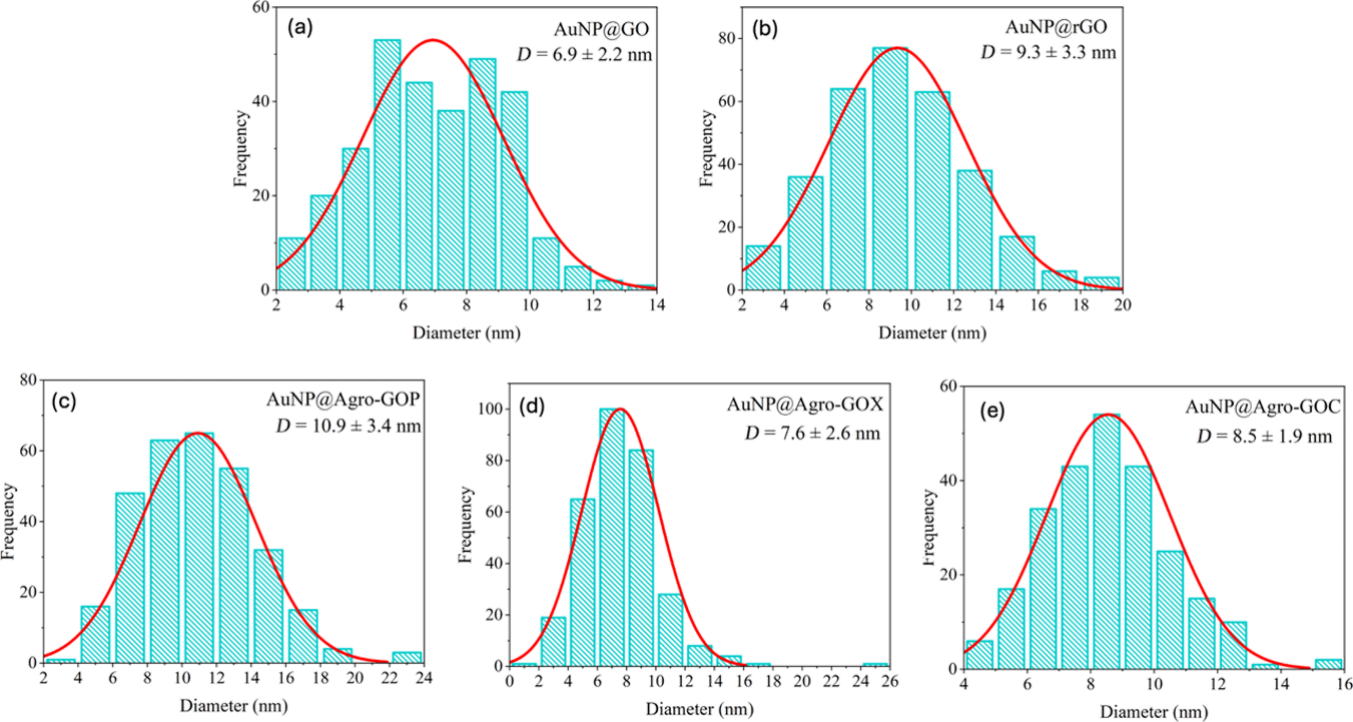
Particle size distribution histograms of AuNPs deposited on GO platforms.

Figure 10 shows TEM micrographs of the AuNP@GO hybrids. Each row corresponds to a specific GO-based hybrid (AuNP@GO, AuNP@rGO, AuNP@Agro-GOP, AuNP@Agro-GOX, and AuNPs@Agro-GOC), and columns show increasing magnifications. AuNP@GO (Figure 10a–d) exhibits a homogeneous distribution of AuNPs with moderate clustering, while preserving the structural integrity of the GO sheets. In contrast, AuNP@rGO (Figure 10e–h) shows pronounced AuNP agglomeration, likely resulting from the lower availability of oxygen-containing functional groups following hydrazine reduction. AuNP@Agro-GOP (Figure 10i–l) shows the most homogeneous and widespread dispersion of AuNPs across the entire sheet surface. This uniform coverage is consistent with the XPS analysis, which revealed that Agro-GOP possesses a large and evenly distributed population of epoxide groups (C4). Because these oxygen functionalities are present throughout the basal plane, not only at edge defects, they provide abundant and readily accessible nucleation sites for AuNP formation, enabling a remarkably uniform nanoparticle distribution. AuNP@Agro-GOX (Figure 10m–p), by comparison, exhibits AuNP deposition predominantly along the sheet edges, where the material appears thinner and more defective. This nucleation at the edges aligns with the XPS results indicating that Agro-GOX is enriched in carbonyl groups (C5), which tend to reside at grain boundaries, defects, and in perimeter regions. As a result, the functional groups in Agro-GOX are mostly available at the borders rather than across the basal surface, limiting AuNP anchoring to the sheet edges and preventing homogeneous dispersion. Finally, AuNP@Agro-GOC (Figure 10q–t) shows a much lower density and greater agglomeration of AuNPs. This poor performance as a nucleation platform can be attributed not only to the lower abundance of reactive oxygen functionalities observed in XPS and ATR-FTIR analyses but also to the specific nature and accessibility of these groups. XPS revealed that Agro-GOC is particularly rich in C–O–C (epoxy/ether) moieties, which exhibit weaker coordination to AuCl_4_^−^ than hydroxy and carboxyl groups. Moreover, residual biomass-derived carbonaceous layers or amorphous carbon deposits may partially mask the GO surface, physically blocking otherwise reactive sites. The combination of these factors reduces the effective density of accessible nucleation sites, resulting in the sparse, agglomerated AuNPs observed in Agro-GOC. The final panel of each row shows high-magnification views of spherical AuNPs with diameters ranging from 6.9 to 10.9 nm, consistent with the particle-size distribution analysis. The average particle sizes were 6.9 ± 2.2 nm (AuNP@GO), 9.3 ± 3.3 nm (AuNP@rGO), 10.9 ± 3.4 nm (AuNP@ Agro-GOP), 7.9 ± 2.6 nm (AuNP@ Agro-GOX), and 8.5 ± 1.9 nm (AuNP@ Agro-GOC) (Figure 11).

These results demonstrate a clear correlation between the surface chemistry of GO-based materials and the nucleation behavior of gold nanoparticles. GO supports with a higher content of oxygen functionalities favor the formation of smaller, more uniformly distributed AuNPs, a feature particularly advantageous for applications in catalysis, sensing, and plasmonic enhancement [30]. In contrast, reduced or less functionalized GO supports lead to the growth of larger and more aggregated particles. Importantly, the green synthesis of GO from agroindustrial waste enables not only control of the overall degree of oxidation but also tuning of specific surface functionalities (e.g., hydroxy, carboxyl, and epoxy groups), as dictated by the nature of the biomass precursor and the processing conditions. This tunability provides a powerful means to tailor the structural and optical properties of AuNP@GO hybrids. Beyond its alignment with environmental sustainability goals, this approach provides a versatile, customizable platform for precisely modulating nanomaterial performance in targeted applications. In addition to the physicochemical characterization, the AuNPs@Agro-GO composites exhibit considerable potential for environmentally oriented technological applications. AuNPs supported on graphene oxide have demonstrated high catalytic efficiency in the reduction of organic pollutants, including nitroaromatic compounds and dye molecules, under mild reaction conditions [57–59]. The synergistic interaction between AuNPs and GO enhances electron transfer, enabling lower noble-metal loading while maintaining high catalytic performance. Furthermore, AuNPs@GO platforms have shown promise in electrochemical and colorimetric sensing of environmental contaminants, allowing for sensitive and rapid detection of toxic species in water [60–62]. Beyond catalysis and sensing, the well-documented antibacterial activity of AuNPs, combined with the large surface area and dispersibility of GO, makes these composites attractive for antimicrobial coatings and water disinfection systems [63–65]. Importantly, the use of agroindustrial waste-derived GO as a carbon precursor adds value to otherwise discarded biomass, offering a low-cost and resource-efficient alternative to conventional graphite sources, while maintaining functional properties suitable for catalytic, sensing, and antibacterial applications. Finally, while AuNP@GO composites are widely reported, most studies rely on conventionally sourced GO, for example obtained by the Hummers' method. In contrast, reports combining Agro-GO with controlled AuNP growth remain scarce. Although Agro-GO may contain a fraction of amorphous carbon, the present results show that it still provides an effective GO-based platform for the controlled growth of AuNPs, underscoring the novelty of the AuNPs@Agro-GO approach.

The present results suggest several directions for future work. In addition to extending the ferrocene-assisted strategy to a broader range of agroindustrial residues, systematic optimization of pyrolysis parameters, particularly temperature and treatment time, will be essential to evaluate their influence on product yield, degree of structural ordering, and the relative contribution of amorphous versus graphitic carbon. Compared to biomass-derived GO prepared via high-temperature carbonization followed by Hummer’s oxidation, the Agro-GO materials synthesized under milder conditions exhibit tunable oxygen functionalization and precursor-dependent modulation of sp^2^-hybridized carbon domains. Such process tuning, combined with optimization of ferrocene loading and post-treatment conditions, could enhance structural reproducibility and facilitate eventual scale-up for industrially relevant applications, where controlled surface chemistry and graphitic domain organization are critical.

## Conclusion

This study demonstrates a sustainable, energy-efficient, and scalable route for transforming agroindustrial waste into GO-based materials and AuNP@GO hybrids. The synthetic strategy, using exceptionally mild carbonization conditions (300 °C, 10 min), substantially reduces energy consumption compared to conventional biomass pyrolysis while still enabling the formation of GO with tunable structure and chemical features. The choice of biomass precursor proved crucial: Agro-GO samples exhibited precursor-dependent oxidation degrees and distinct distributions of oxygen-containing functional groups, as evidenced by UV–vis, Raman, FTIR, and XPS. TEM imaging further highlighted pronounced morphological differences among the Agro-GO samples, ranging from compact, defect-rich aggregates to partially layered domains. These differences in surface chemistry and defect structure directly govern the photochemical growth of AuNPs, with highly oxidized and functionalized matrices promoting the formation of smaller, well-dispersed nanoparticles. In contrast, less oxidized or more graphitic supports yield larger, more aggregated AuNPs. Overall, the ability to tailor GO properties by carefully selecting agricultural precursors provides a versatile pathway for designing application-specific carbon–metal hybrids within a circular-economy framework.

## Supporting Information

File 1 Additional experimental data.

## Data Availability

All data that supports the findings of this study is available in the published article and/or the supporting information of this article.
