# Supplementary material for: Upcycling agroindustrial waste into graphene oxide supports for gold nanoparticles: toward sustainable nanomaterials
Source: Beilstein J Nanotechnol. 2026 Apr 1;17:489–504. doi: 10.3762/bjnano.17.32 (PMC13058278; doi:10.3762/bjnano.17.32)
Supplement: File 1 — Additional experimental data. [file Beilstein_J_Nanotechnol-17-489-s001.pdf]

BEILSTEIN JOURNAL OF NANOTECHNOLOGY

Supporting Information

for

Upcycling agroindustrial waste into graphene oxide supports for gold nanoparticles: toward sustainable nanomaterials

Juan Marcos Castro-Tapia, Selene Acosta, Hiram Joazet Ojeda-Galván, Elsie Evelyn Araujo-Palomo, Edgar Giovanni Villabona-Leal and Mildred Quintana

Beilstein J. Nanotechnol. **2026,** *17,* 489–504. doi:10.3762/bjnano.17.32

Additional experimental data

License and Terms: This is a supporting information file under the terms of the Creative Commons Attribution License (https://creativecommons.org/licenses/by/4.0). Please note that the reuse, redistribution and reproduction in particular requires that the author(s) and source are credited and that individual graphics may be subject to special legal provisions.
The license is subject to the *Beilstein Journal of Nanotechnology* terms and conditions: (https://www.beilstein-journals.org/bjnano/terms)

O 1s XPS spectra of GO, rGO, Agro-GOP, Agro-GOX, and Agro-GOC are shown in Figure S1. Four components labeled as O1, O2, O3, and O4 are reported. O1 (530 eV) is assigned to C=O, O2 (531 eV) to (C=O)-OH, O3 (532 eV) corresponds to C–O bonding, and O4 (533.0 eV) to C–O–C [1-3].

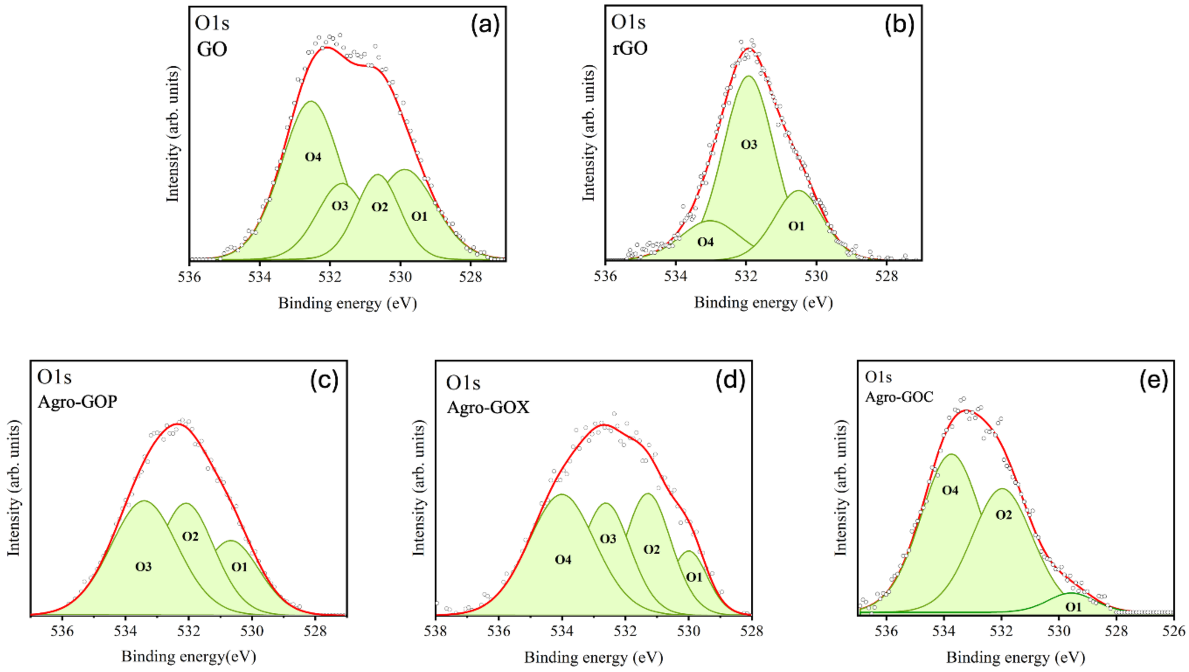

Figure S1: O 1s XPS spectra of (a) GO, (b) rGO, (c) Agro-GOP, (d) Agro-GOX, (e) Agro-GOC.

Table S1: Relative area of the components in the O 1s analysis spectra for GO samples.

	Relative area (%)			
**sample**	**O1**<br>**530 eV**<br>**C=O**	**O2**<br>**531 eV**<br>**(C=O)-OH**	**O3**<br>**532 eV**<br>**C–O**	**O4**<br>**533.0 eV**<br>**C–O–C**
**GO**	26.9	14.3	15.3	43.5
**rGO**	22.3	0	62.2	15.5
**Agro-GOP**	23.4	34.6	42.0	0
**Agro-GOX**	11.0	26.3	25.5	37.2
**Agro-GOC**	8.3	35.0	0	56.0

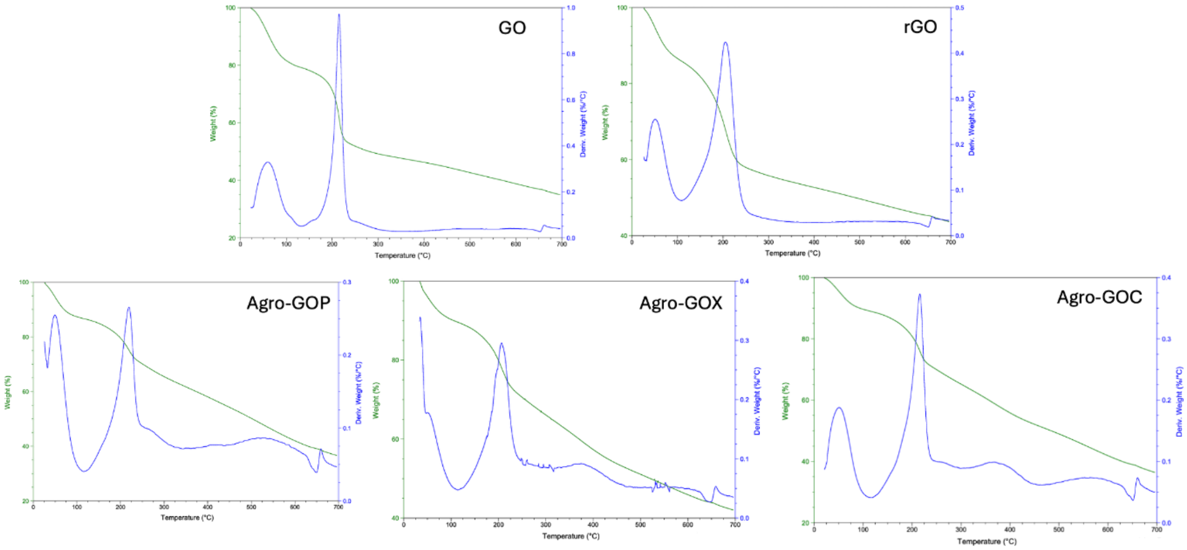

Figure S2: Derivative thermogravimetric (DTG) curves of GO, rGO, and agro-derived samples (Agro-GOP, Agro-GOX, and Agro-GOC).

References

1. Kwan, Y. C. G.; Ng, G. M.; Huan, C. H. A. *Thin Solid Films* **2015,** *590,* 40–48. doi:10.1016/j.tsf.2015.07.051
2. Sun, P.; Wang, Y.; Liu, H.; Wang, K.; Wu, D.; Xu, Z.; Zhu, H. *PLoS One* **2014,** *9,* e111908. doi:10.1371/journal.pone.0111908
3. Mattevi, C.; Eda, G.; Agnoli, S.; Miller, S.; Mkhoyan, K. A.; Celik, O.; Mastrogiovanni, D.; Granozzi, G.; Garfunkel, E.; Chhowalla, M. *Adv. Funct. Mater.* **2009,** *19,* 2577–2583. doi:10.1002/adfm.200900166
